# Supplementary material for: Ultrahigh resistance of hexagonal boron nitride to mineral scale formation
Source: Nat Commun. 2022 Aug 4;13:4523. doi: 10.1038/s41467-022-32193-4 (PMC9352771; doi:10.1038/s41467-022-32193-4)
Supplement: Supplementary file 1 — Supplementary Information [file 41467_2022_32193_MOESM1_ESM.pdf]

Supplementary Information

Ultrahigh resistance of hexagonal boron nitride to mineral scale formation

Kuichang Zuo et al.

Supplementary Note 1

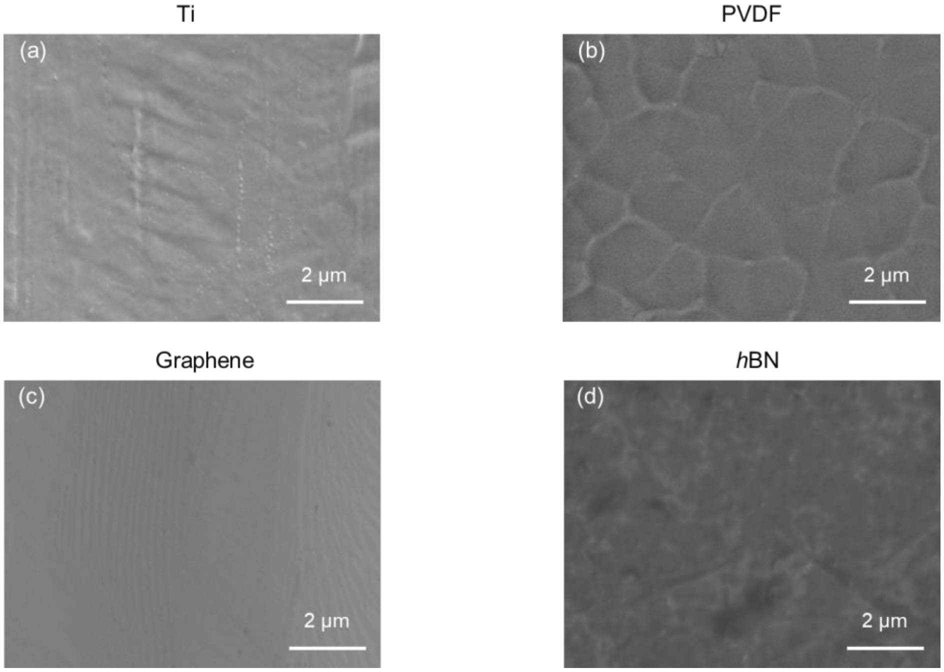

Supplementary Figure 1. Surface morphology of (a) Ti, (b) PVDF, (c) graphene, and (d) *h*BN characterized by SEM.

After synthesis, graphene and *h*BN films were transferred to SiO_2/Si substrates using a PMMA-assisted transfer method and characterized by Raman spectroscopy, AFM, and XPS. In this method, a PMMA film was first spin-coated on the graphene or *h*BN grown on Cu foil. The sample was then immersed in a $FeCl_3$ solution to dissolve the Cu substrate. The graphene or *h*BN immobilized on the PMMA film was then transferred to DI water, and subsequently collected onto a SiO_2/Si substrate with the graphene or *h*BN side attaching to the substrate surface. Finally, acetone and IPA were used to dissolve the PMMA layer, exposing the graphene or *h*BN surface. The prepared graphene- or *h*BN-coated SiO_2/Si was dried at 60 ^{o}C and stored at room temperature before characterization.

Raman spectra in Supplementary Figure 2a shows the G band and the 2D band. The high 2D/G intensity ratio and invisible D band indicate the graphene is a high-quality single layer. Supplementary Figure 2b presents E_{2g} Raman peak of *h*BN. The AFM data in Supplementary Figure 2c show that the thickness of the *h*BN film is ~ 1.7 nm, which is about 4-layer thick. High-resolution XPS spectra of B 1s and N 1s are demonstrated in Supplementary Figure 2d and 2e, respectively. The B/N atomic ratio was calculated to be ~1.04.

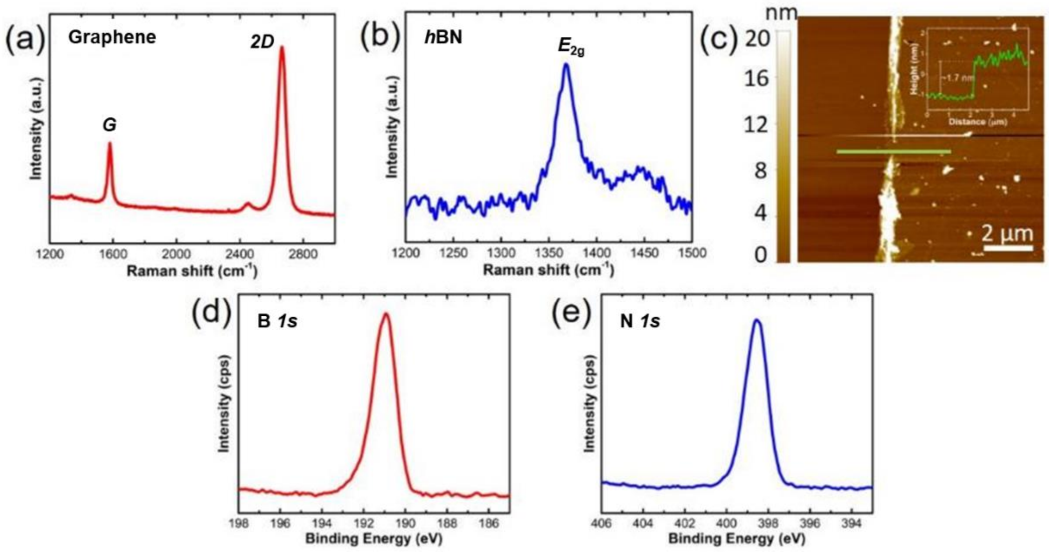

Supplementary Figure 2. Characterization of graphene and *h*BN transferred on SiO_2/Si. Raman spectra of (a) graphene and (b) *h*BN. (c) AFM image of *h*BN film and height profile along the green line. High-resolution XPS spectra of (d) B 1s and (e) N 1s of *h*BN.

Supplementary Note 2

The induction time for homogeneous nucleation in the supersaturated $CaCO_3$ solution (vaterite SI of 0.075) was determined by dynamic light scattering (DLS) measurements using a NanoBrook Omni (Brookhaven Instrument, Holtsville, NY). DLS data in Supplementary Figure 3 showed that the induction time for homogeneous nucleation of the prepared supersaturated $CaCO_3$ solution is longer than 20 min, before which the light scattering intensity was low and stable, indicating no formation of $CaCO_3$ crystals.

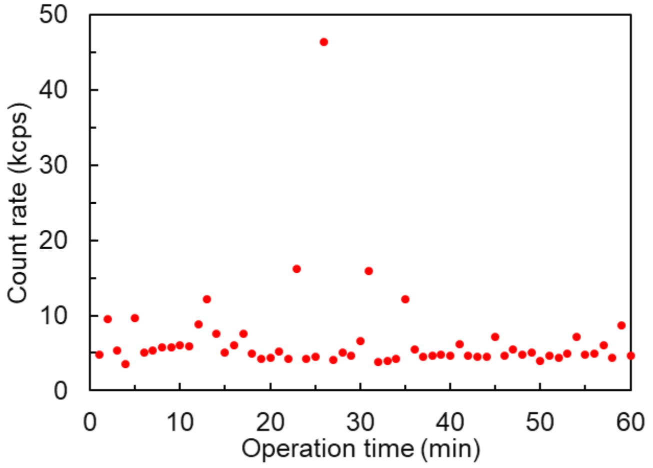

Supplementary Figure 3. Induction time of the supersaturated $CaCO_3$ solution.

After heterogeneous nucleation experiments, the sample surfaces were imaged by SEM. Supplementary Figure 4 shows the PVDF surface after heterogeneous nucleation experiments. Mineral crystals can be clearly seen grown from the surface. Similar

phenomena are also observed on the Ti, *h*BN, and graphene surfaces as well. As shown in the figure, vaterite with hexagonal or semi-hexagonal structure is the main specie formed on the surface, other species such as calcite and aragonite are not found.

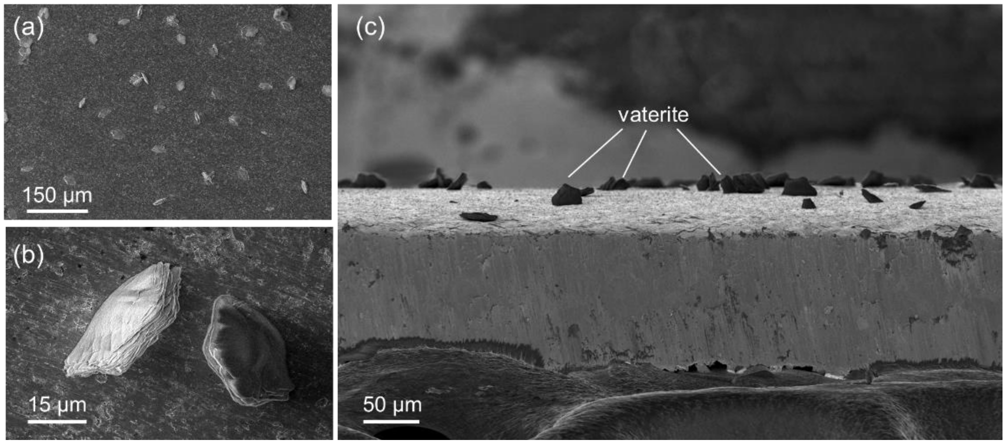

Supplementary Figure 4. Vaterite with hexagonal or semi-hexagonal structure is the main specie grown on the PVDF surface. (a) Top view SEM image; (b) Magnified top view SEM image illustrates that hexagonal or semi-hexagonal vaterite is "grown from" the surface, evidence of heterogeneous nucleation; (c) Sectional view of the crystal-grown surface shows that vaterite is grown from the surface.

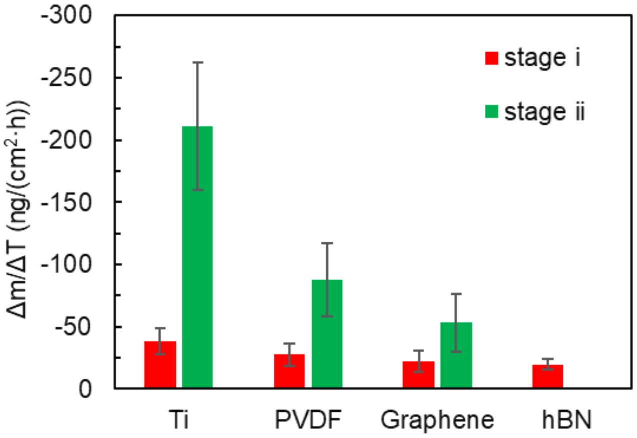

Supplementary Figure 5. Mass increase rates on various surfaces during the nucleation and crystal growth stages of $CaCO_3$ scale formation. Error bars represent the standard deviation of corresponding results.

Supplementary Note 3

The nanotip can push the grown vaterite crystal at a direction perpendicular (Supplementary Figure 6a, Supplementary Movie 2) or parallel (Supplementary Figure 6b, Supplementary Movie 3) to the plane of the semi-hexagonal vaterite crystal. However, the obtained results are greatly different (Supplementary Figure 6a4, b4), this is because the anisotropy structure of the vaterite crystal. As shown in Supplementary Figure 6a1, when pushing the crystal at the perpendicular direction, the obtained force

can be greatly affected by the pushing position (distance from the contact position to the substrate). Therefore, to obtain reliable results, data reported in the manuscript are all obtained by pushing the vaterite crystal with a parallel direction to its plane (Supplementary Figure 6b).

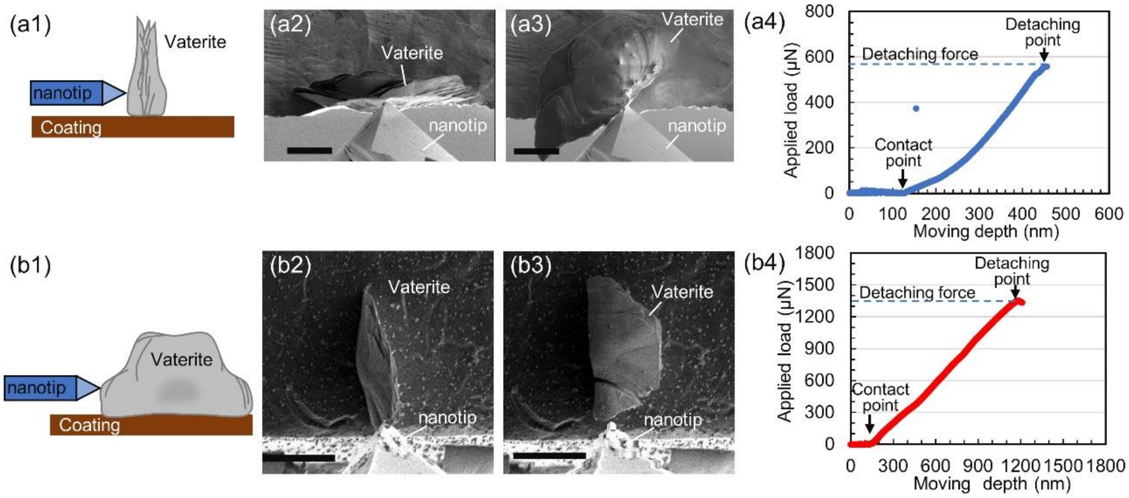

Supplementary Figure 6. Testing the binding force between vaterite crystal and the underlying coating. (a) Nanoindenter tip is perpendicular to the plane of a vaterite crystal. (a1) Sectional view schematic; Top view SEM image of the vaterite crystal (a2) before and (a3) after being pushed off from the underlying coating; (a4) Applied load increases linearly with the displacement of the nanoindenter tip upon contact with the vaterite crystal. (b) Nanoindenter tip is parallel to the plane of a vaterite crystal. (b1) Sectional view schematic; Top view SEM image of the vaterite crystal (b2) before and (b3) after being pushed off from the underlying coating; (b4) Applied force in the parallel direction is much higher than that obtained in the perpendicular direction. The scale bars in a2, a3, b2 and b3 represent 10 µm.

Supplementary Note 4

(1) Characteristics of hBN and graphene:

It is important to understand the physical and chemical characteristics of *h*BN and graphene in order to understand their behavior in contact with water and other molecules. We created two flakes of each material containing 180 atoms (including the hydrogen atoms that were used to passivate the flakes). The geometry optimization of both flakes was first performed using the Hartree-Fock based PM6 method[1], followed by a final geometry optimization with density functional theory (DFT), with the Becke three-parameter Lee-Yang-Parr exchange-correlation functional (B3LYP) and 6-31G(d) basis set functions. These simulations were performed using MOPAC2016[2] and GAUSSIAN09[3] software. In all geometry optimizations, the Grimme-D3 method[4] was used to consider corrections for nonbonded interactions (van der Waals). The resulting optimized structures are presented in Supplementary Figure 7. We have analyzed the central region of the flakes to minimize the edge effects.

For graphene, the carbon atoms are arranged in the sp^2 hybridization, in which each carbon makes 3 σ bonds in plane and one π with the remaining p_z orbital, giving rise to the delocalized π system in graphene. As for *h*BN, the N and B atoms also have sp^2 hybridization. However, the hybridization of B results in an empty p_z orbital, which shares the electrons of the lone pair (orbital p_z) of N to create a π bond. So, it is expected that some differences in charge distribution occur in each system.

After the geometry optimization, both graphene and *h*BN flakes exhibit a planar configuration, as expected. The differences in the flake properties are clear due to the intrinsic characteristic of their component (C, B, and N). From the frontier electronic orbitals of each case in Supplementary Figure 7 (highest occupied and lowest unoccupied molecular orbitals, HOMO and LUMO, respectively), we see very delocalized orbitals for graphene due to its delocalized π system. As for *h*BN, the HOMO orbital is more localized at the N atoms, while the LUMO on the B atoms. This effect results from the nature of the p_z orbitals and the difference in electronegativity of B and N: N shares its lone pair electrons thus N is more electronegative than B, the electrons of the π system tend to be more localized on the N atoms. As a result, the almost empty p_z orbital of B tends to have an important contribution to the LUMO orbital.

We presented the charge distribution (Mulliken charges) on the surface of each material. As shown in Supplementary Figure 7, the charge at the center of the graphene is uniform, resulting in a small dipole moment (0.036 D). *h*BN exhibits a different pattern: B remains more positive while N is more negative, resulting in a greater dipole moment of 0.047 D. Supplementary Figure 7 also shows the electrostatic potential distribution for both materials, which indicates the attractive (towards red) and repulsive (towards blue) regions. The C atoms in graphene has an electronegativity of 2.55, while B and N on *h*BN have an electronegativity of 2.04 and 3.04, respectively, which results in a difference on the surface electron distribution.

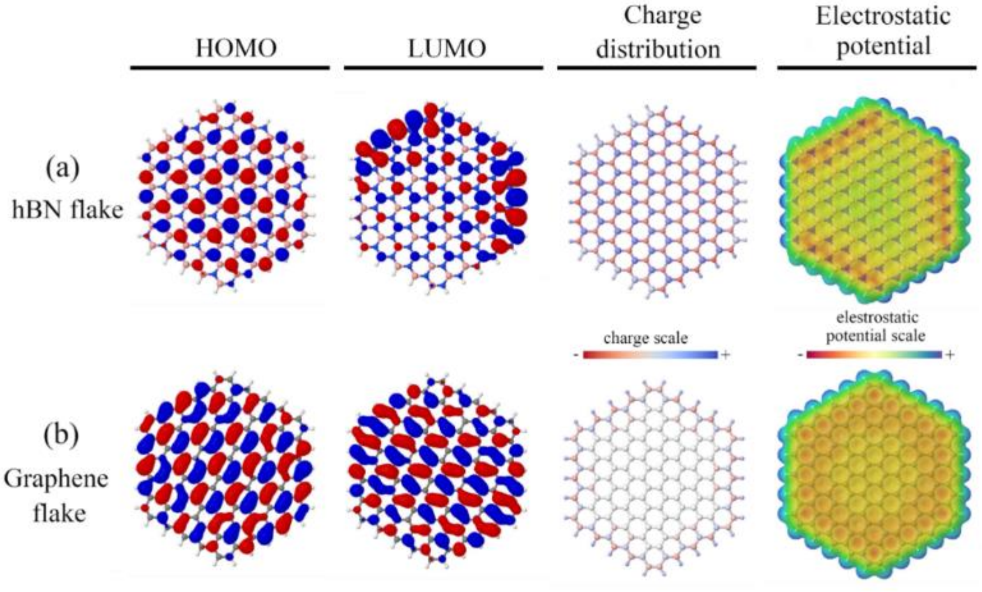

Supplementary Figure 7. Spatial distribution of the HOMO, LUMO, charge (Mulliken), and the electrostatic potential of (a) *h*BN and (b) graphene flakes.

(2) Interaction between water molecules and hBN or graphene:

Since water molecules are polar, they can induce structural deformations and/or charge redistribution on graphene and *h*BN surface. Therefore, we investigated the graphene and *h*BN behavior when they contact with water molecules. Using the optimized geometries obtained before, we randomly added 26 water molecules 1.4 Å away from basal plane of each surface, the geometry was reoptimized in a PM6-D3 followed by a DFT-D3/B3LYP/6-31G(d) theory level. The resulting optimized systems are presented in Supplementary Figure 8.

As shown Supplementary Figure 8, there is a small deformation of both graphene and *h*BN flakes due to the presence of water molecules. The *h*BN flake becomes concave with ~7.2° of deformation from planarity, while graphene flake deviates ~5.0° from planarity. This is an indication that the interaction of water molecules with graphene is weaker than with *h*BN. The strong interaction with *h*BN can be explained by the polarity of water and the charge differences in B and N atoms, which does not occur in graphene, as discussed in details below.

Water molecules are more densely packed on the *h*BN surface: they are arranged in approximately 2 layers, with a circular diameter of ~10.5 Å; the closest water molecules are located around 2.92 Å from the flake surface and ~2.45-2.65 Å from each other in the first layer. The average distance of a water molecule from *h*BN is in agreement with quantum Monte Carlo simulations[5]. For the graphene, the water molecules are arranged mostly in one layer (only 3 molecules out of this layer), with a circular diameter of ~12.3 Å; the closest water molecules are around 2.95 Å from the flake and ~2.55-2.65 Å from each other in the first layer.

It is also seen that the presence of the water molecules does not significantly modify the HOMO distribution, which remains essentially the same. The charge distribution is also similar to that without water, but the dipole moment changed to 8.1 D and 5.3 D for *h*BN and graphene flakes, respectively, due to the structural distortions.

These results suggest that the water molecules are denser and stay closer to the *h*BN than graphene surface. The alternating negative and positive regions on the *h*BN surface promote interactions with the positive and negative portions of water molecules, respectively. The negative regions on *h*BN attract the H atoms in water, and the positive regions attract O atoms, resulting in an increased attraction of water molecules and a bigger flake distortion on the *h*BN surface than on the graphene. As a comparison, the graphene surface does not have alternative negative and positive regions, therefore it has a less attraction towards water molecules.

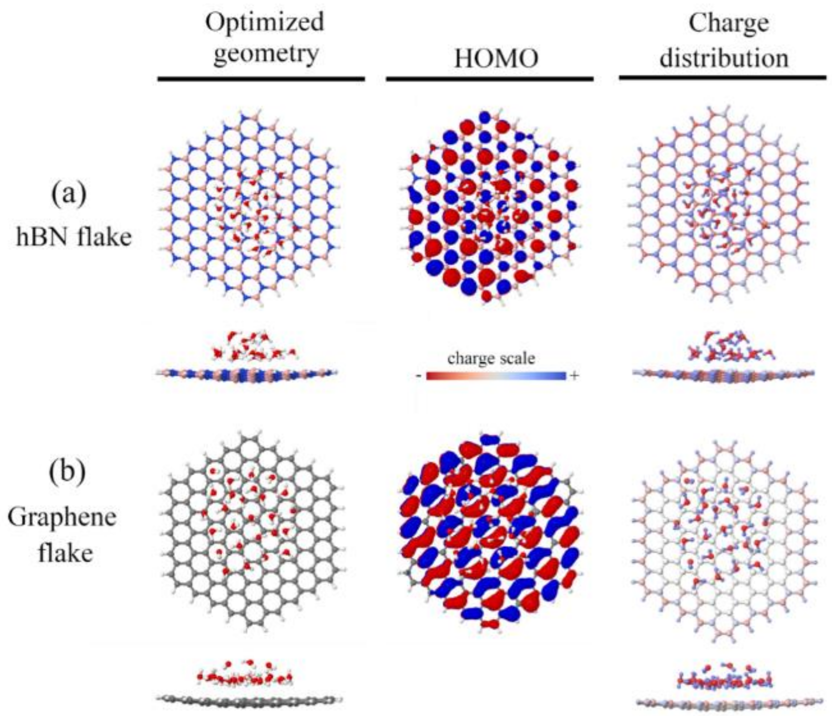

Supplementary Figure 8. Optimized geometries, HOMO orbitals, and charge distribution on (a) *h*BN and (b) graphene surface with the presence of water molecules.

(3) Interactions between hBN/graphene and non-hydrated Ca^{2+}, CO_3^{2-}, $CaCO_3$:

After analyzing the interactions between water and *h*BN and graphene surfaces, we investigated the interaction of Ca^{2+}, CO_3^{2-}, and $CaCO_3$ with the *h*BN and graphene surfaces. This step is important as it allows us to elucidate the role of water by comparing these calculations with those in the presence of water. We placed the Ca^{2+}, CO_3^{2-}, and $CaCO_3$ around 1.4 Å away from the center of the *h*BN or graphene surface, and performed a geometry optimization of these systems (in a PM6-D3 followed by a DFT-D3/B3LYP/6-31G(d) theory level). The resulting structures are shown in Supplementary Figure 9.

When Ca^{2+} is introduced on the *h*BN surface, it forms bonds with two nitrogen atoms with a bond length of 2.3 Å, shorter than previous experimental result (Ca-N bond length of ~2.5 Å) because of the N-Ca-N double bond formed in this study. This is expected because the electrons accumulated on N atoms. As a comparison, Ca^{2+} stays close to the center of the hexagonal carbon ring on graphene surface, with a distance of ~1.65 Å from each carbon atom. Since the graphene surface has a delocalized electron cloud, Ca^{2+} does not favor specific carbon atoms. Flake deformation is observed on both *h*BN and graphene surfaces, and an energy of ~9.5 eV is needed to remove the Ca^{2+} from both surfaces.

In the case of CO_3^{2-}, we see that its edges (O atoms) are more negative than the center (C atom), despite that one of the O atoms is less negative compared to the others. Therefore, it is expected that the O atoms are attracted by the positive regions of *h*BN flake. As shown in Supplementary Figure 9, the two most negative O atoms interact with

B, forming bonds with a length of ~1.56 Å, slightly longer than the experimentally measured bond length (~1.4 Å). This may be attributed to the neighboring N atoms that are negatively charged and cause electrostatic repulsion to the O atoms. For graphene, we observe that the least negative O atom prefers to make a bond with a carbon atom, with a bond length of 1.41 Å and a good agreement with the experimental length for a single C-O bond (1.43 Å). We also observe deformation of the flakes due to these bonds; the energy needed to remove CO_3^{2-} from the surface is ~7.2 and 6.6 eV for *h*BN and graphene, respectively.

With $CaCO_3$, we see a deficit of electrons in Ca and C (positive), while the O atoms remain more negative; it is also observed that the O atom to the right is less negative than the other two (Supplementary Figure 9). On the *h*BN surface, we observe that Ca bonds with an N atom, with a bond length of 2.3 Å. On the graphene surface, there is no bond formation; $CaCO_3$ aligns itself with the less negative O atom closest to the graphene surface, and the molecule stays at ~3.3 Å from the graphene surface. The flakes have almost no deformation and the necessary energy to remove the $CaCO_3$ from the surface is relatively low, 1.5 and 0.1 eV for *h*BN and graphene, respectively.

In general, these simulations suggest that these chemical species will interact with both *h*BN and graphene flakes, and all species have stronger interactions with *h*BN than graphene.

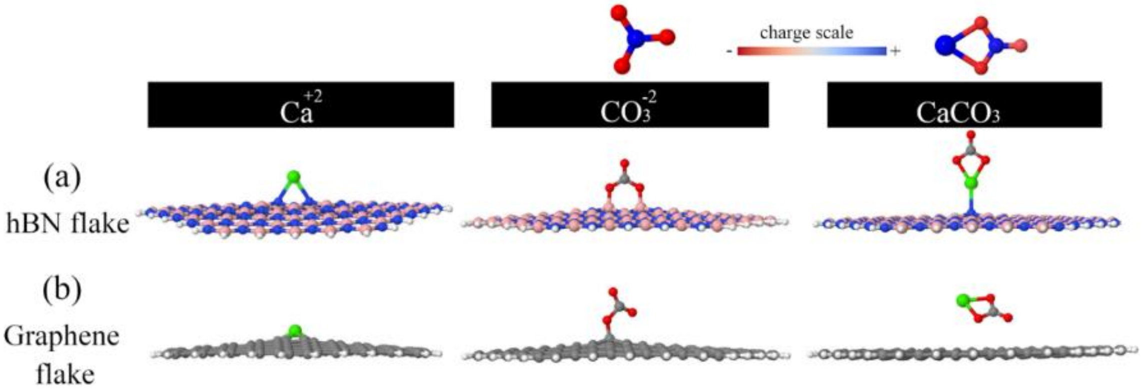

Supplementary Figure 9. Optimized geometries of the Ca^{2+}, CO_3^{2-}, and $CaCO_3$ in contact with (a) *h*BN and (b) graphene surfaces.

(4) Interaction between hBN/graphene and the hydrated Ca^{2+}, CO_3^{2-}, and $CaCO_3$:

To investigate the interactions between Ca^{2+}, CO_3^{2-}, $CaCO_3$ and *h*BN and graphene with the presence of water, we first performed Molecular Dynamics (MD) Simulation using the LAMMPS software[6] to evaluate the number of water molecules that are carried by Ca^{2+}, CO_3^{2-}, and $CaCO_3$. We created a box of 20 × 20 × 20 $Å^3$ and added Ca^{2+}, CO_3^{2-}, or $CaCO_3$ at the center of the box surrounded by 50 water molecules, as shown in Supplementary Figure 10a, 10b, and 10c below. Then, we allow the system to freely evolve at room temperature and 1 atm for 2 ns in ReaxFF force field[7] (time-step of 0.1 fs, NPT ensemble, and periodic boundary conditions). We then evaluated the pair radial

distribution (g(r)) and obtained the number of the water molecules in the first shell enclosing the Ca^{2+}, CO_3^{2-}, or $CaCO_3$.

Supplementary Figure 10d presented the g(r) between the Ca^{2+} and water molecules (the distances between Ca^{2+} and the oxygen atoms of water). The first peak shows that the closest water molecules are 2.1 Å from Ca^{2+}. The coordination number (CN) was found to be 6 from the area under the first peak of the g(r) curve, i.e., 6 water molecules remain in the first shell surrounding Ca^{2+}, as shown in the inset of Supplementary Figure 10d. These results agree with data found in the literature.

A CN of 2 was found for CO_3^{2-}, with water molecules 2.3 Å away from CO_3^{2-} (Supplementary Figure 10e). For $CaCO_3$ (Supplementary Figure 10f), g(r) was calculated for both Ca^{2+} and CO_3^{2-} in the molecule. We found a CN of 5 and 2 for constituents Ca^{2+} and CO_3^{2-}, respectively, showing This a total of 7 water molecules in the first shell surrounding the $CaCO_3$.

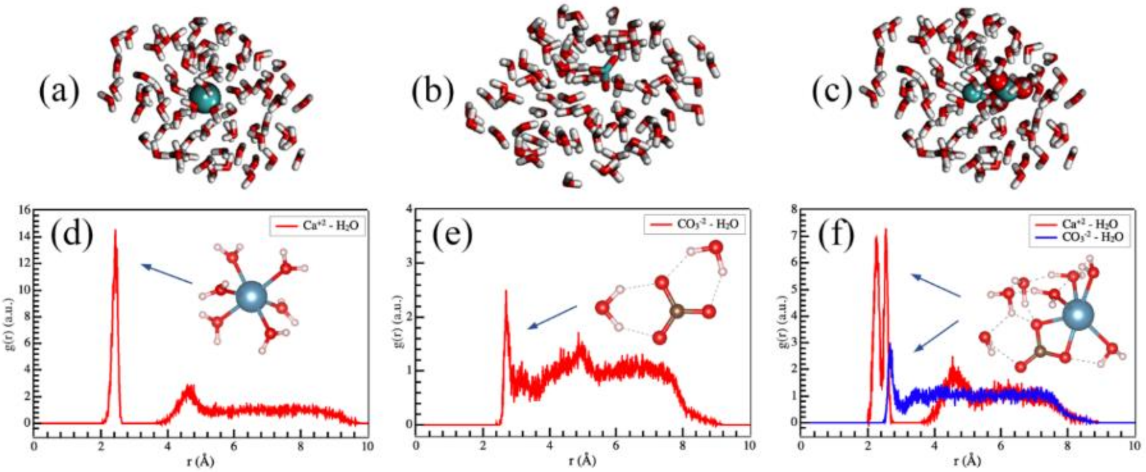

Supplementary Figure 10. Initial configurations of (a) Ca^{2+}, (b) CO_3^{2-}, and (c) $CaCO_3$, respectively, enclosed by 50 water molecules. The corresponding pair radial distribution (g(r)) in relation to the water molecules evolution for 2ns at room temperature for (d) Ca^{2+}, (e) CO_3^{2-}, and (f) $CaCO_3$.

We evaluate the interaction of hydrated Ca^{2+}, CO_3^{2-} and $CaCO_3$ with *h*BN and graphene surfaces in the presence of water. The hydrated species, Ca^{2+}(6 H_2O), CO_3^{2-}(2 H_2O), and $CaCO_3$(7 H_2O) were positioned on the *h*BN and graphene surfaces, and another 26 water molecules were added to the system. We optimize the geometry of these systems (in a PM6-D3 followed by a DFT-D3/B3LYP/6-31G(d) theory level) and analyze the conformation and final distances between Ca^{2+}, CO_3^{2-}, and $CaCO_3$ from the surfaces and water molecules. Supplementary Figure 11 shows the optimized configurations. It can be seen that the Ca^{2+}, CO_3^{2-}, and $CaCO_3$ stay above the closest water layer on the *h*BN and graphene surface in all cases, suggesting that it is not energetically favorable for them to diffuse through the water molecules.

When the hydrated Ca^{2+} is added in the *h*BN system, we observe that water molecules form almost two layers and remain close to the flake. The distance among water molecules ranges from 2.45 to 2.65 Å, and those in the first layer have the smallest

intermolecular distance. The distance between the first water layer and the *h*BN surface is ~2.97 Å. There are 7 closest water molecules surrounding Ca^{2+}, 1 more than in the absence of the *h*BN flake; the distance between the closest water and Ca^{2+} also increased from 2.1 Å in the absence of *h*BN to ~2.35 Å with *h*BN. As a result, Ca^{2+} is 5.15 Å away from the *h*BN surface. On the graphene surface, water molecules also formed almost two layers, with an intermolecular distance ranging from 2.55 to 2.65 Å; water in the first layer has the smallest intermolecular distance. Eight water molecules surround Ca^{2+}, with the closest water molecules at ~2.45 Å. Ca^{2+} is ~4.96 Å away from the graphene surface. These results show that water molecules form a denser structure on the surface of *h*BN than on graphene, leading to a larger distance between Ca^{2+} and the surface. Considering that the distance of the first layer water molecules is smaller on *h*BN (2.45 Å) than on graphene (2.55 Å), Ca^{2+} diffusion through the water molecules to reach the *h*BN surface is expected to be less energetically favorable.

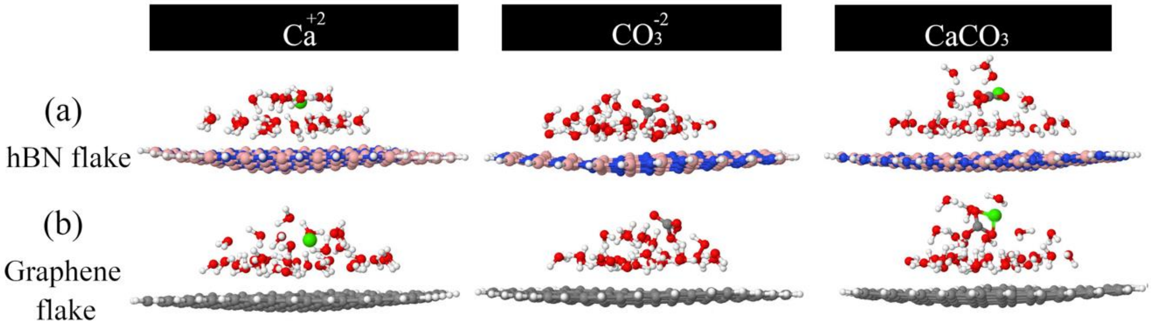

Supplementary Figure 11. Optimized geometries of the Ca^{2+}, CO_3^{2-}, and $CaCO_3$ in contact with (a) *h*BN, and (b) graphene surfaces with the presence of water.

For CO_3^{2-} in the *h*BN system, water molecules remain 2.35-2.65 Å from each other, with water in the first layer has the smallest intermolecular distance. The closest layer of water molecules to the *h*BN surface is ~2.65 Å away from the surface, and the water molecules closest to CO_3^{2-} is at ~2.65 Å. The CO_3^{2-} ion remains 4.85 Å away from the *h*BN surface. Doing the same analysis for graphene, the water molecules remain in a range of 2.55-2.65 Å from each other. In the graphene system, the closest water molecules are ~2.75 Å from the graphene surface, and the closest water molecules to CO_3^{2-} is ~2.65 Å. The CO_3^{2-} remains 4.85 Å away from the graphene flake. These results also confirm that water molecules are more densely packed near *h*BN surface while CO_3^{2-} stays at a comparable distance from the *h*BN and graphene surfaces.

In the $CaCO_3$ and *h*BN system, water molecules are 2.45-2.65 Å from each other. The closest water molecules to the *h*BN flake are at ~2.90 Å and the closest water molecules to $CaCO_3$ are at ~2.45 Å. The $CaCO_3$ remains 5.15 Å away from the *h*BN surface. For the corresponding graphene, the water molecules remain in a range of 2.45-2.65 Å from each other. In comparison, the closest water molecules are at ~2.92 Å from the graphene surface and ~2.45 Å from $CaCO_3$. $CaCO_3$ remains 5.05 Å away from the graphene surface.

From these results, we come to the following conclusions: (i) water molecules stay closer to *h*BN than to graphene; (ii) the first layer of water molecules are denser on *h*BN

than graphene. The combination of (i) and (ii) indicates that it will be more difficult for chemical species to diffuse through the water layer on *h*BN than that on graphene.

In order to test this hypothesis, we performed an exploratory simulation using the Nudge Elastic Band (NEB) method[8] to determine the minimum energy for a Ca^{2+} ion to move through the closest layer of water molecules on *h*BN and graphene surfaces. The NEB method looks for a favorable path with minimum energy requirement for the Ca^{2+} ion to penetrate the water layer. As shown in Figure 4F in the manuscript, the necessary energy increases significantly with the approaching of Ca^{2+} to the water layer; 1.0 eV more energy is needed for Ca^{2+} to penetrate the water layer on *h*BN than on graphene. The absolute values of the energy barrier and energy barrier difference may not be accurate because the hydration water molecules on the *h*BN and graphene surfaces are set as "frozen", i.e., immobile, in the simulation. This may result in higher calculated energy barriers. However, the qualitative trend should remain the same: it is more difficult for Ca^{2+} to reach the *h*BN surface than the graphene surface.

Supplementary Table 1. Lattice constants for various materials at 300 K[9-11].

**Material**	**Lattice constant (Å)**	**Crystal structure**	**Material**	**Lattice constant (Å)**	**Crystal structure**
C (diamond)	3.567	Diamond (FCC)	CsF	6.02	Halite
C (graphite)	a = 2.461<br>c = 6.708	Hexagonal	CsCl	4.123	Caesium chloride
Si	5.431020511	Diamond (FCC)	CsI	4.567	Caesium chloride
Ge	5.658	Diamond (FCC)	Al	4.046	FCC
AlAs	5.6605	Zinc blende (FCC)	Fe	2.856	BCC
AlP	5.4510	Zinc blende (FCC)	Ni	3.499	FCC
AlSb	6.1355	Zinc blende (FCC)	Cu	3.597	FCC
GaP	5.4505	Zinc blende (FCC)	Mo	3.142	BCC
GaAs	5.653	Zinc blende (FCC)	Pd	3.859	FCC
GaSb	6.0959	Zinc blende (FCC)	Ag	4.079	FCC
InP	5.869	Zinc blende (FCC)	W	3.155	BCC
InAs	6.0583	Zinc blende (FCC)	Pt	3.912	FCC
InSb	6.479	Zinc blende (FCC)	Au	4.065	FCC
MgO	4.212	Halite (FCC)	Pb	4.920	FCC
SiC	a = 3.086<br>c = 10.053	Wurtzite	V	3.0399	BCC
CdS	5.8320	Zinc blende (FCC)	Nb	3.3008	BCC
CdSe	6.050	Zinc blende (FCC)	Ta	3.3058	BCC
CdTe	6.482	Zinc blende (FCC)	TiN	4.249	Halite
ZnO	a = 3.25<br>c = 5.2	Wurtzite (HCP)	ZrN	4.577	Halite
ZnO	4.580	Halite (FCC)	HfN	4.392	Halite
ZnS	5.420	Zinc blende (FCC)	VN	4.136	Halite
PbS	5.9362	Halite (FCC)	CrN	4.149	Halite
PbTe	6.4620	Halite (FCC)	NbN	4.392	Halite
BN	a = 2.504<br>c =6.661	Hexagonal	TiC	4.328	Halite
	3.6150	Zinc blende (FCC)			

BP	4.5380	Zinc blende (FCC)	$ZrC_{0.97}$	4.698	Halite
CdS	*a* = 4.160<br>*c* = 6.756	Wurtzite	$HfC_{0.99}$	4.640	Halite
ZnS	*a* = 3.82<br>*c* = 6.26	Wurtzite	$VC_{0.97}$	4.166	Halite
AlN	*a* = 3.112<br>*c* = 4.982	Wurtzite	$NC_{0.99}$	4.470	Halite
GaN	*a* = 3.189<br>*c* = 5.185	Wurtzite	$TaC_{0.99}$	4.456	Halite
InN	*a* = 3.533<br>*c* = 5.693	Wurtzite	Cr_3C_2	*a* = 11.47<br>*b* = 5.545<br>*c* = 2.830	Orthorombic
LiF	4.03	Halite	WC	*a* = 2.906<br>*c* = 2.837	Hexagonal
LiCl	5.14	Halite	ScN	4.52	Halite
LiBr	5.50	Halite	$LiNbO_3$	*a* = 5.1483<br>*c* = 13.8631	Hexagonal
LiI	6.01	Halite	$KTaO_3$	3.9885	Cubic perovskite
NaF	4.63	Halite	$BaTiO_3$	*a* = 3.994<br>*c* = 4.034	Tetragonal perovskite
NaCl	5.64	Halite	$SrTiO_3$	3.98805	Cubic perovskite
NaBr	5.97	Halite	$CaTiO_3$	*a* = 5.381<br>*b* = 5.443<br>*c* = 7.645	Orthorhombic perovskite
NaI	6.47	Halite	$PbTiO_3$	*a* = 3.904<br>*c* = 4.152	Tetragonal perovskite
KF	5.34	Halite	$EuTiO_3$	7.810	Cubic perovskite
KCl	6.29	Halite	$SrVO_3$	3.838	Cubic perovskite
KBr	6.60	Halite	$CaVO_3$	3.767	Cubic perovskite
KI	7.07	Halite	$BaMnO_3$	*a* = 5.673<br>*c* = 4.71	Hexagonal
RbF	5.65	Halite	$CaMnO_3$	*a* = 5.27<br>*b* = 5.275<br>*c* = 7.464	Orthorhombic perovskite
RbCl	6.59	Halite	$SrRuO_3$	*a* = 5.53<br>*b* = 5.57<br>*c* = 7.85	Orthorhombic perovskite
RbBr	6.89	Halite	$YAlO_3$	*a* = 5.179<br>*b* = 5.329<br>*c* = 7.37	Orthorhombic perovskite
RbI	7.35	Halite			

Supplementary Note 5

Supplementary Table 2. Composition of produced water from oil & gas industry.

		Concentration (mg/L)
Main cations	Na^+	38621.2 ± 988.9
	Ca^{2+}	3976.3 ± 233.7

	Mg^{2+}	624.7 ± 9.0
	Sr^{2+}	406.5 ± 8.5
	K^{+}	304.5 ± 79.2
	Fe	222.5 ± 145.0
	Mn	18.0 ± 12.0
	Zn^{2+}	16.7 ± 10.9
	Cr	5.0 ± 2.0
	Ba^{2+}	4.0 ± 1.0
	Al^{3+}	ND
	Ag^{+}	ND
	Cd	ND
	Co^{2+}	ND
	Cu^{2+}	ND
	Ni^{2+}	ND
	Pb^{2+}	ND
Main anions	Cl^{-}	70631.4 ± 615.9
	NO_3^{-}	2033.8 ± 109.1
	SO_4^{2-}	732.4 ± 34.1
	PO_4^{3-}	2980.1 ± 7.2

ND = not detected

Supplementary References:

1 Stewart, J. J. P. Optimization of parameters for semiempirical methods V: Modification of NDDO approximations and application to 70 elements. *J. Mol. Model.* **13**, 1173-1213 (2007).

2 MOPAC2016 (Stewart Computational Chemistry, Colorado Springs, CO, USA, 2016).

3 Gaussian 09, Revision D.01 (Gaussian, Inc., Wallingford CT, 2009).

4 Grimme, S., Antony, J., Ehrlich, S. & Krieg, H. A consistent and accurate ab initio parametrization of density functional dispersion correction (DFT-D) for the 94 elements H-Pu. *J. Chem. Phys.* **132** (2010).

5 Wu, Y., Wagner, L. K. & Aluru, N. R. Hexagonal boron nitride and water interaction parameters. *The Journal of Chemical Physics* **144**, 164118 (2016).

6 Plimpton, S. Fast Parallel Algorithms for Short-Range Molecular-Dynamics. *J. Comput. Phys.* **117**, 1-19 (1995).

7 Pitman, M. C. & van Duin, A. C. T. Dynamics of Confined Reactive Water in Smectite Clay-Zeolite Composites. *J. Am. Chem. Soc.* **134**, 3042-3053 (2012).

8 Jonsson, H., Mills, G. & Jacobsen, K. W. in *Classical and Quantum Dynamics in Condensed Phase Simulations* 385-404.

9 Saha, B., Acharya, J., Sands, T. D. & Waghmare, U. V. Electronic structure, phonons, and thermal properties of ScN, ZrN, and HfN: A first-principles study. *J. Appl. Phys.* **107**, 033715 (2010).

10 Davey, W. P. Precision Measurements of the Lattice Constants of Twelve Common Metals. *Physical Review* **25**, 753-761 (1925).

11 Part A · Table 2, Part 1: Datasheet from Landolt-Börnstein - Group III Condensed Matter · Volume 4A: "Part A" in SpringerMaterials (https://doi.org/10.1007/10201420_50) (Springer-Verlag Berlin Heidelberg).
